# Association of Urogenital Symptoms with History of Water Contact in Young Women in Areas Endemic for *S. haematobium*. A Cross-Sectional Study in Rural South Africa

**DOI:** 10.3390/ijerph13111135

**Published:** 2016-11-14

**Authors:** Hashini Nilushika Galappaththi-Arachchige, Ingrid Elise Amlie Hegertun, Sigve Holmen, Erik Qvigstad, Elisabeth Kleppa, Motshedisi Sebitloane, Patricia Doris Ndhlovu, Birgitte Jyding Vennervald, Svein Gunnar Gundersen, Myra Taylor, Eyrun Floerecke Kjetland

**Affiliations:** 1 Norwegian Centre for Imported and Tropical Diseases, Department of Infectious Diseases Ullevaal, Oslo University Hospital, Postboks 4956 Nydalen, Oslo 0450, Norway; ingrid.hegertun@gmail.com (I.E.A.H.); sigve.holmen@gmail.com (S.H.); elisabethkleppa@gmail.com (E.K.); e.f.kjetland@medisin.uio.no (E.F.K.); 2 Institute of Clinical Medicine, University of Oslo, Oslo 0312, Norway; UXERQU@ous-hf.no; 3 Department of Gynaecology, Women and Children’s Division, Ullevaal University Hospital, Oslo 0450, Norway; 4 Discipline of Obstetrics and Gynaecology, Nelson R Mandela School of Medicine, University of KwaZulu-Natal, Durban 4001, South Africa; sebitloanem@ukzn.ac.za; 5 Imperial College London, Claybrook Center, London W68LN, UK; p.ndhlovu@imperial.ac.uk; 6 Section for Parasitology and Aquatic Diseases, Faculty of Health and Medical Sciences, University of Copenhagen, Copenhagen 2200, Denmark; bjv@sund.ku.dk; 7 Research Unit, Sorlandet Hospital, Kristiansand 4615, Norway; svein.g.gundersen@sshf.no; 8 Department of Global Development and Planning, University of Agder, Kristiansand 4630, Norway; 9 Discipline of Public Health Medicine, Nelson R Mandela School of Medicine, College of Health Sciences, University of KwaZulu-Natal, Durban 4001, South Africa; taylor@ukzn.ac.za

**Keywords:** *Schistosoma haematobium*, female genital schistosomiasis, water contact, urogenital symptoms, sexually transmitted infections

## Abstract

Female genital schistosomiasis is a neglected tropical disease caused by *Schistosoma haematobium*. Infected females may suffer from symptoms mimicking sexually transmitted infections. We explored if self-reported history of unsafe water contact could be used as a simple predictor of genital schistosomiasis. In a cross-sectional study in rural South Africa, 883 sexually active women aged 16–22 years were included. Questions were asked about urogenital symptoms and water contact history. Urine samples were tested for *S. haematobium* ova. A score based on self-reported water contact was calculated and the association with symptoms was explored while adjusting for other genital infections using multivariable logistic regression analyses. *S. haematobium* ova were detected in the urine of 30.5% of subjects. Having ova in the urine was associated with the water contact score (*p* < 0.001). Symptoms that were associated with water contact included burning sensation in the genitals (*p* = 0.005), spot bleeding (*p* = 0.012), abnormal discharge smell (*p* = 0.018), bloody discharge (*p* = 0.020), genital ulcer (*p* = 0.038), red urine (*p* < 0.001), stress incontinence (*p* = 0.001) and lower abdominal pain (*p* = 0.028). In *S. haematobium* endemic areas, self-reported water contact was strongly associated with urogenital symptoms. In low-resource settings, a simple history including risk of water contact behaviour can serve as an indicator of urogenital schistosomiasis.

## 1. Introduction

It is estimated that more than 112 million people worldwide are infected with the freshwater parasite *Schistosoma haematobium* [1]. *S. haematobium* is thought to be the main etiological agent of urogenital schistosomiasis [2]. This disease is mostly endemic in Sub-Saharan Africa, the Middle East and most recently an outbreak was reported in Corsica, France [3,4]. The disease is prevalent in poor communities without access to safe water and proper sanitation [5]. Rural communities that rely on agriculture and fishing are especially at risk as well as women and children who are using rivers for their daily chores or recreational activities [5].

Infection with *S. haematobium* occurs through contact with contaminated freshwater containing the larval form of the parasite (cercariae), which is released by the *Bulinus africanus* snail [6]. The cercariae penetrate the skin, enter the bloodstream and follow it to the liver [6]. Here, they mature and mate before they migrate as male and female couples to the venous plexa surrounding the urogenital organs where the female starts producing ova [6]. Some of these ova are released back into the environment through excreta while some get lodged in the urogenital organs [6]. The urogenital morbidity seen with *S. haematobium* infection is largely due to the host’s immune response to the schistosome ova [6].

Female genital schistosomiasis (FGS) is defined as schistosomiasis affecting the female reproductive tract with characteristic lesions such as grainy sandy patches, homogenous yellow patches, rubbery papules and abnormal blood vessels [7]. FGS has been shown to be associated with infertility, miscarriage, extrauterine pregnancies, urinary symptoms and genital symptoms mimicking sexually transmitted infections (STIs) [2]. Furthermore, recent studies have shown that FGS may make women more susceptible to human immunodeficiency virus (HIV) [8,9]. This might be due to due to local inflammation of the genital mucosa, breach of the mucosal barrier and immunological changes in the mucosa [8,9].

Several studies have found an association between water contact and schistosomiasis and have shown that a simple questionnaire on water contact could be used to identify urogenital schistosomiasis in *S. haematobium* endemic areas [10,11,12]. To our knowledge, there has only been one study that explored the association between genital symptoms and water contact in an *S. haematobium* endemic area [13]. However, this was carried out in young girls 10–12 years of age, prior to becoming sexually active. In *S. haematobium* endemic areas with limited resources FGS is an important differential diagnosis to venereal disease and cervical cancer [2]. This study seeks to explore self-reported water contact as a simple predictor for urogenital symptoms in young women living in a rural schistosomiasis endemic area in South Africa.

## 2. Materials and Methods

### 2.1. Study Area

A cross-sectional study was performed in schools in three districts in KwaZulu-Natal, on the east coast of South Africa. According to the South African census of 2011, in KwaZulu-Natal 63.6% of the households had access to piped water in their dwelling or yard, 22.4% had access to piped water on a communal stand and 14.1% had no access to protected water. The subtropical climate in this region ranges from very hot, rainy summers (November–February) to cool and dry winters (June–August). The province is endemic for *S. haematobium* [13,14]. We targeted schools with more than 300 pupils situated in rural areas in Ilembe, Uthungulu and Ugu districts below the altitude of 300 m above sea level as a sampling frame, and we randomly selected 70 schools for the study. Based on one urine sample, 31 out of 70 schools with a prevalence of more than 20% schistosomiasis were included.

### 2.2. Recruiting Participants

The recruiting took place from 2011 to 2013. Included schools were visited and informed about schistosomiasis. All female students above the age of 16 were invited to the information sessions. Sampled by convenience, sexually active and consenting young women aged 16–22 years were invited for gynaecological examinations. Pregnant women, virgins and non-consenting women were excluded. Dates for possible investigations were provided by the teachers and subsequently discussed with the women individually.

### 2.3. Questionnaire

Trained research assistants performed the interviews in their local language (isiZulu) using a questionnaire. Answers were translated into English. Questions were asked about current or previous symptoms, such as bloody discharge, spot bleeding, genital itch, burning sensation in the genital area, painful intercourse, lower abdominal pain, upper abdominal pain, abnormal discharge smell and colour, red urine, pain on urination, and stress incontinence. The questionnaire also included questions about previous treatment with Praziquantel (the standard treatment for schistosomiasis).

Rivers, dams, lakes, streams and ponds were defined as unsafe water sources. Detailed questions were asked about lifetime exposure to the most common water-related activities in the study area (laundry, washing blankets, playing, personal hygiene, crossing, collection of water for domestic use, and fishing) [13,15]. Participants were shown a diagram of the human body and were asked to describe how much of their body got exposed to water during the different water contact activities. The percentage of the body exposure was recorded according to the rule of nines (commonly used in body burn charts) [16]. Each water activity frequency could be graded from a maximum score of four if they had used it “daily” to zero if they had “never” used the water body for the specific activity.

### 2.4. Water Contact Score

A water contact score was created for each individual based on the frequency of each activity, duration of unsafe water contact, percentage of the body exposed and total number of years of unsafe water contact during their lifetime.

As shown in Table 1, we quantified the estimated risk involved with each of the activities by creating an activity risk coefficient (ARC), which was calculated based on the reported average duration of water contact and reported percentage of the body exposed [15].

**Figure 1:**
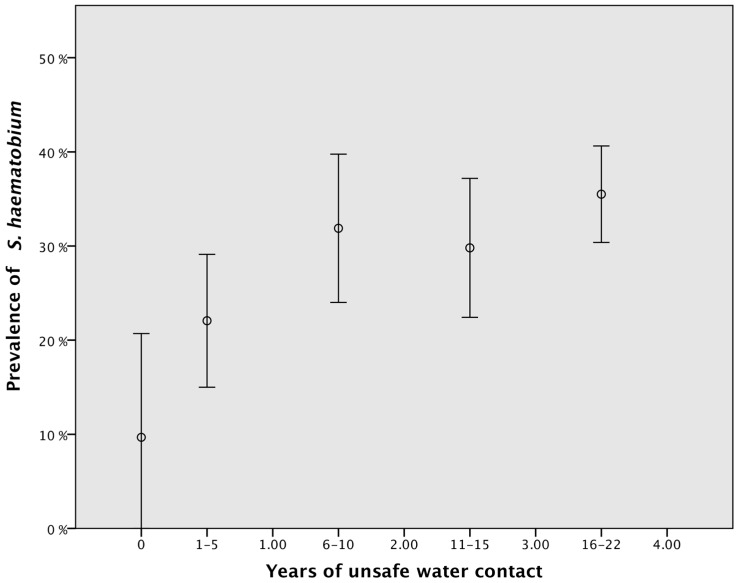
Prevalence of *S. haematobium* ova in the urine and total number of years of unsafe water contact. Error bars: 95% confidence interval (CI).

Figure 1 shows that the prevalence of schistosomiasis increases gradually with the increased number of years of water contact, up to six years of water contact, after which the prevalence plateaus. Based on the number of years exposed to unsafe water, we assigned a lifetime exposure risk coefficient (ERC) from 0 to 2, where 0 was assigned to those participants who reported no water contact, 1 was assigned to those who reported having water contact for 1–5 years and 2 was assigned to those who reported having had unsafe water contact for more than six years.

The compound water score was calculated for each individual by multiplying the frequency of water contact (F) by each of the activities’ risk coefficients (ARC) and by the lifetime exposure risk coefficient (ERC):

Water contact score = F × ARC × ERC

The water contact score was normalized to a range of 0–10 for ease of presentation.

### 2.5. Sample Collection and Laboratory Analyses

Consenting participants were invited for a gynaecological examination, which included collection of cervico-vaginal lavage (CVL) for STI analyses and Papanicolaou (Pap) smears [17]. One urine sample was collected from each participant between 10 a.m. and 2 p.m. [18]. For quality control purposes, two 10 mL urine samples were prepared for microscopy as previously described [14]. The presence of at least one ovum was defined as a positive diagnosis. Blood was collected (30 mL) in sterile acid-citrate-dextrose anti-coagulated Vacutainer tubes (Becton, Dickinson and Company (BD), Franklin Lakes, NJ, USA) [19].

*Neisseria gonorrhoea* and *Chlamydia trachomatis* were detected using a strand displacement assay (ProbeTec CT/GC, BD) [20]. In-house PCR (Laboratory of Infection, Prevention and Control, UKZN, Durban, South Africa) was used for *Trichomonas vaginalis* [20]. Bacterial vaginosis was diagnosed using Nugent’s Score [21]. A score above seven was considered a positive diagnosis [22]. Syphilis screening was performed using rapid plasma reagin (RPR, Macro Vue test 110/112, BD), and positive tests were confirmed using *Treponema pallidum* hemagglutination assay (TPHA, Omega Diagnostics Group PLC, Alva, Scotland, UK) [23]. *Herpes simplex* type 2 antibodies were detected in serum using ELISA (Ridascreen HSV 2 IgG, Davies Diagnostics, Randburg, South Africa) [24]. All serology was done on serum samples frozen at −80 °C. *Candida albicans* was detected in the Pap smear by microscopy [25,26].

### 2.6. Ethical Considerations

All participants signed individual, informed, written consent forms. The participants were made aware of their right to withdraw at any time during the study. The study was approved by the Biomedical Research Ethics Committee (BREC), University of KwaZulu-Natal (Ref BF029/07), KwaZulu-Natal Department of Health (Reference HRKM010-08) and the Regional Committee for Medical and Health Research Ethics (REC), South Eastern Norway (Ref 469-07066a1.2007.535). The Departments of Health and Education in Ugu, Ilembe and Uthungulu districts, KwaZulu-Natal, gave permissions for this study. The ethical committees, BREC (annual renewal) and REC, were aware that minors, aged 16 and 17 were participating in the study and specifically approved independent minor consent without parental consent. We followed the South African syndromic protocol to diagnose and treat findings at the point of care. Once the laboratory results were available, patients were contacted for further management as required. Praziquantel was offered to all participants as part of a Department of Health mass drug administration campaign.

### 2.7. Statistical Analyses

Statistical analyses were done using Statistical Package for Social Sciences (SPSS) version 22 (IBM, Chicago, IL, USA). Graphs were made using Prism version 6 (Graphpad, San Diego, CA, USA). Univariable logistic regression was used to evaluate the association between water contact frequency, self-reported symptoms and genital infection by calculating the odds ratio (OR) and the corresponding 95% confidence intervals (CI). Variables that were associated within a 15% significance level were included in the multivariable logistic regression analyses (MVA). Age was included in the MVA regardless of the significance level as this is considered an important socio-biological confounder.

## 3. Results

### 3.1. General Characteristics of the Study Population

**Figure 2:**
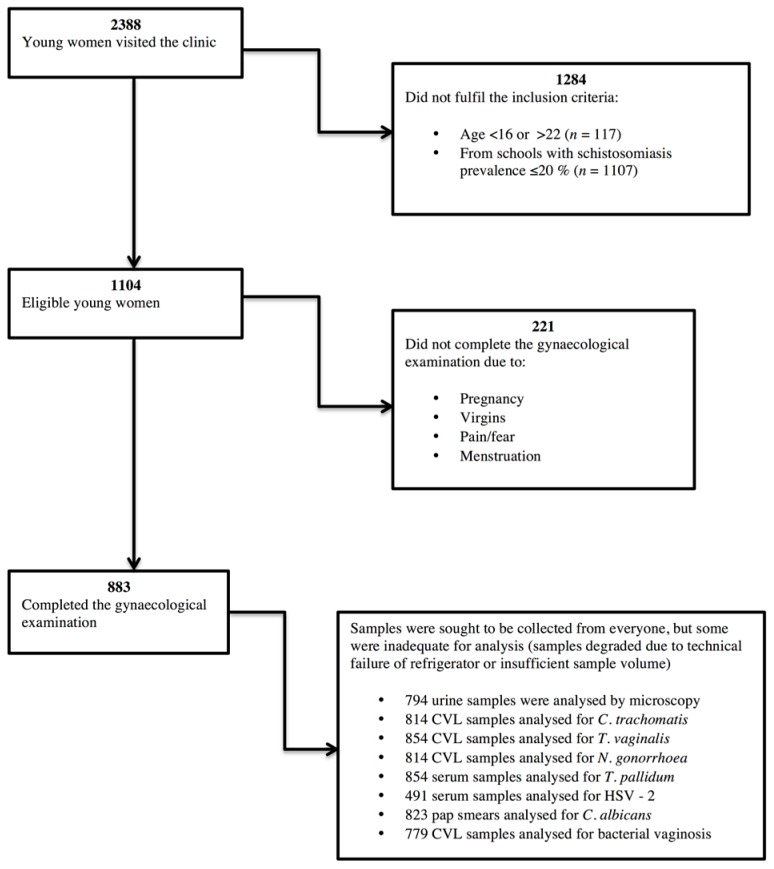
Flowchart of the participants and sample selection procedure. CVL: cervico-vaginal lavage.

A flowchart showing the process of including study participants is presented in Figure 2. The age distribution within the study population is presented in Table 2. A total of 883 sexually active young women aged 16–22 years (median age 19 years) were included in the study. Out of these included participants, 794 individuals provided one urine sample for microscopy. *S. haematobium* ova were detected in 30.5% (242/794). Only 25.3% (223/882) had received Praziquantel treatment prior to participating in this study and 31.2% (275/882) did not know whether they had ever received treatment.

**Figure 3:**
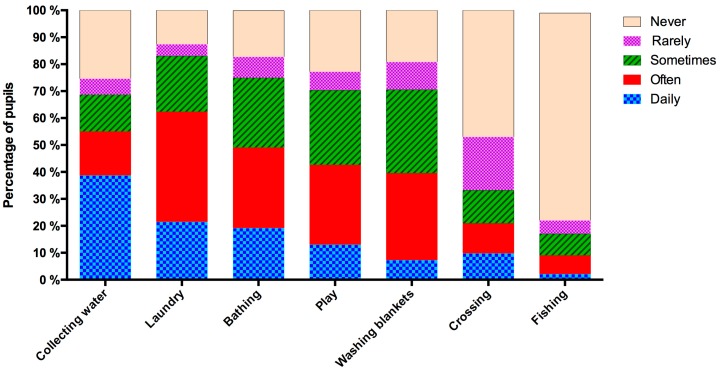
Frequency whereby the participants engaged in different water contact activities in their lifetime, *n* = 883.

**Figure 4:**
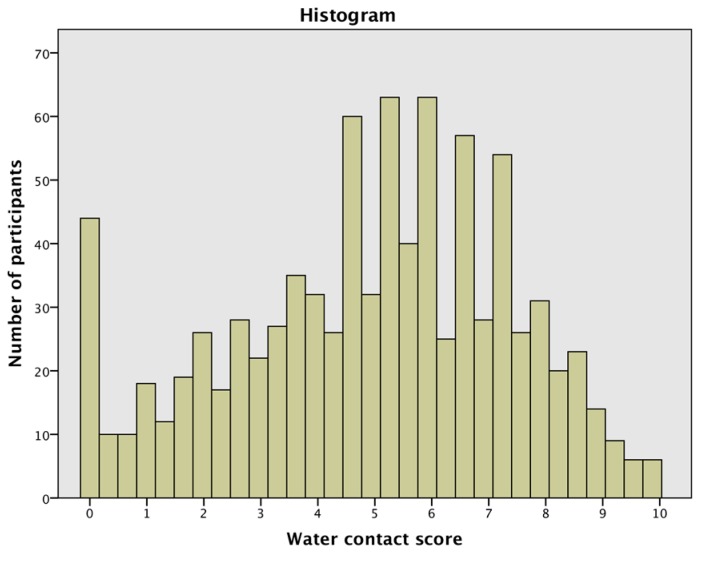
Distribution of the calculated water contact score. The score was calculated as the multiplicative product of frequency of water contact, a risk coefficient assigned to the each of seven common water contact activities and a risk coefficient assigned to the number of years exposed to unsafe water. Mean = 4.94, standard deviation = 2.424, *n* = 883.

Almost all participants, 95.7% (845/883), reported having had unsafe water contact at some time in their lives. Of these, 94.0% (830/883) reported having had unsafe water contact for domestic purposes and 77.2% (682/883) for recreation. The most common unsafe water contact source was rivers (87.8%, 740/843). More than half of the participants, 52.0% (459/883), reported having current unsafe water contact. Figure 3 shows how often the participants engaged in the different water contact activities. Figure 4 shows the distribution of the calculated water contact score in the population.

**Figure 5:**
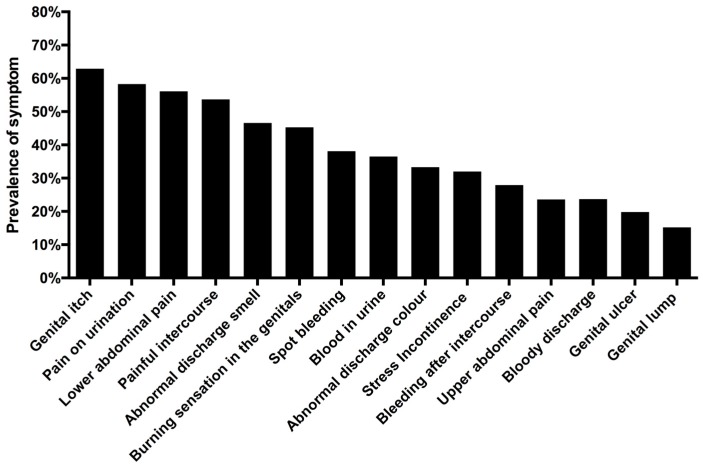
Urogenital symptoms (lifetime occurrence).

The pupils’ median sexual debut age was 16 years (range 10–21). Participants had a median of two lifetime partners (range 1–13). *C. trachomatis* was the most common STI with a prevalence of 26.7% (217/814), followed by *T. vaginalis* (19.8%, 169/853) and *N. gonorrhoea* (12.0%, 98/814). Syphilis was the least common STI in this study group with a prevalence of 2.1% (18/858). *C. albicans* was detected in 13.5% (111/823) and bacterial vaginosis in 62.1% (484/779). The prevalence of self-reported gynaecological and urinary symptoms is shown in Figure 5.

### 3.2. Water Contact and Urinary Schistosomiasis

**Figure 6:**
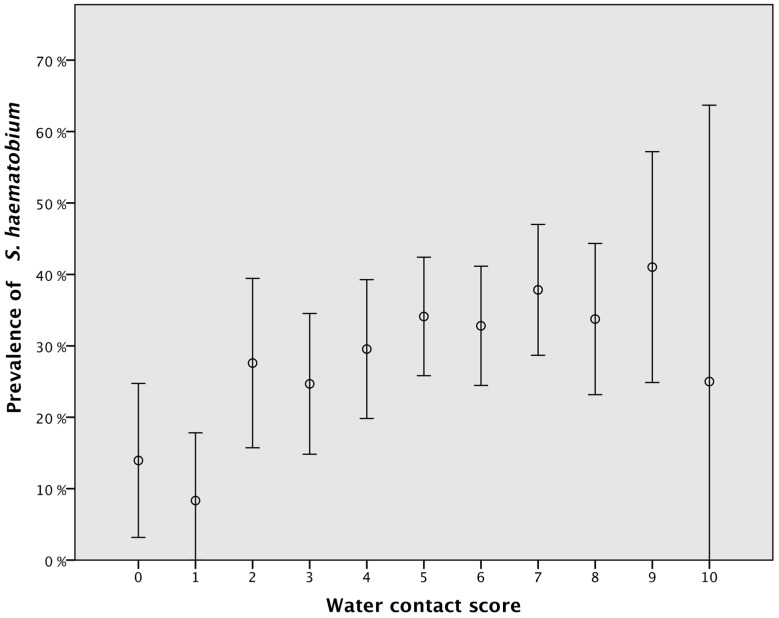
The prevalence of *S. haematobium* ova in the urine increases with increasing water contact score. Error bars: 95% CI.

Having *S. haematobium* ova in the urine was significantly associated with the calculated water contact score (OR 1.15, 95% CI: 1.07–1.22, *p* < 0.001). Figure 6 shows that there is a gradual increase in urinary *S. haematobium* prevalence with an increase of the water contact score. We detected *S. haematobium* ova in the urine of 14% of those who denied any water contact (score zero). There were only eight participants with the maximum score of 10.

### 3.3. Unsafe Water Contact and Self-Reported Urogenital Symptoms

Self-reported symptoms that were significantly associated with lifetime water contact score (controlling for age and for genital infections) are presented in Table 3. The water contact score was not associated with bleeding after intercourse (adjusted (adj.) OR 1.08, 95% CI: 1.00–1.17, *p* = 0.059), painful intercourse (adj. OR 1.05, 95% CI: 1.00–1.11, *p* = 0.092), abnormal discharge colour (adj. OR 1.05, 95% CI: 0.99–1.12, *p* = 0.090), genital itch (adj. OR 1.05, 95% CI: 0.99–1.09, *p* = 0.102), genital lumps (adj. OR 1.00, 95% CI: 0.92–1.08, *p* = 0.964), pain on urination (adj. OR = 1.05, 95% CI: 0.99–1.11, *p* = 0.123) and upper abdominal pain (adj. OR 1.01, 95% CI: 0.94–1.08, *p* = 0.786). As shown in the Figure 5, post-coital bleeding, upper abdominal pain and genital lumps are amongst the least common symptoms, whereas genital itch, dyspareunia and dysuria are very common.

### 3.4. Current Unsafe Water Contact and Urogenital Symptoms

Table 4 shows a subsample of participants who were currently reporting using unsafe water. Several symptoms that were associated with lifetime unsafe water contact were found to not be associated with current unsafe water contact; these were bloody discharge (adj. OR 1.00, 95% CI: 0.88–1.15, *p* = 0.963), lower abdominal pain (adj. OR 1.08, 95% Cl: 0.98–1.20, *p* = 0.137) and genital ulcers (adj. OR 1.08, 95% CI: 0.95–1.21, *p* = 0.238). The following symptoms were not associated when analysing for current water contact: abnormal discharge colour (adj. OR 1.10, 95% CI: 1.00–1.22, *p* = 0.061), painful intercourse (adj. OR 1.09, 95% CI: 0.88–1.15, *p* = 0.084) genital itch (adj. OR 1.02, 95% CI: 0.92–1.13, *p* = 0.717), genital lump (adj. OR 1.07, 95% CI: 0.92–1.24, *p* = 0.380) and upper abdominal pain (adj. OR 1.03, 95% CI: 0.91–1.16, *p* = 0.677).

## 4. Discussion

In a young population of women living in an *S. haematobium* endemic area of KwaZulu-Natal, South Africa, we found an association between self-reported unsafe water contact and urogenital schistosomiasis in young women (age 16–22). Our findings further confirm that there is significant association between unsafe water contact and genital symptoms, even after adjusting for sexually transmitted infections.

When analysing a subsample of young women, who at the time of attending the clinic, reported to be currently relying on unsafe water contact, we found a difference in symptoms compared to the group reporting on total lifetime exposure to unsafe water. Those with current water contact reported bleeding after intercourse and painful urination, but not bloody discharge, genital ulcers and lower abdominal pain, which were symptoms associated with total lifetime unsafe water contact. This may suggest that the presentations of schistosomiasis are complex and possibly different throughout life. Furthermore, in a chronic stage of the disease, although there may not be live worms left, old calcified eggs may still be lodged in the mucosa, causing inflammation and recruitment of CD4 positive cells [27].

In the same study area, Hegertun et al found an association between genital symptoms and water contact in schoolgirls aged 10–12 years indicating that infection with *S. haematobium* starts in early childhood [13]. To date, we have found only six studies that have explored the association between gynaecological symptoms and schistosomiasis [13,28,29,30,31,32]. Those studies were small and from different countries and age groups. Interestingly, the studies have also shown other urogenital symptoms to be associated with *S. haematobium* infection (e.g., genital itch). In *S. haematobium* endemic areas, Kjetland et al. found that *S. haematobium* may be the most common cause of genital morbidity and mucosal lesions [7]. Based on these findings, we argue that in *S. haematobium* endemic areas, urogenital schistosomiasis should be considered as a differential diagnosis in all women presenting with symptoms of sexually transmitted diseases.

More than half of the participants were still using an unsafe water source at the time of the interview, mainly for domestic chores. Only a quarter of the participants reported having received Praziquantel treatment at some point in their life. Furthermore, South Africa has still not implemented a national schistosomiasis control and prevention programme. The current study area is categorized as moderately endemic for schistosomiasis according to the World Health Organization (prevalence of 10%–50%) [33]. Our findings indicate that there is an urgent need for public health initiatives to educate policy makers, health care professionals as well as those who are at risk.

### Limitations

There is a day-to-day variation in ova excretion and by collecting samples over several days, we would have found a higher prevalence of *S. haematobium* [18]. The water bodies that have been used by the participants have not been checked for *Bulinus africanus* snails shedding *S. haematobium* cercaria. All the data we have gathered on symptoms and water contact history are self-reported. Although the participants were unaware of their schistosomiasis status we cannot preclude recall bias. Furthermore, the inaccuracy of self-reported water contact is evident from the fact that the *S. haematobium* prevalence was 14% in those who denied having had unsafe water contact. Combining the questionnaire with direct observational data could have improved the accuracy of this study [34].

The research clinic aimed at being youth-friendly and the learners were informed that female genital schistosomiasis can be asymptomatic. However, those experiencing symptoms might be more likely to participate in order to receive treatment [35]. Therefore, the symptoms reported by the study population might not be truly representative of the study area. Cultural factors may have also played a role; such as fear of being stigmatized, which may have lead to under-reporting of symptoms. Lastly, cultural and psychological factors and chronic disease could have influenced their perception of symptoms.

## 5. Conclusions

In an *S. haematobium* endemic area, unsafe water contact was associated with urogenital symptoms in teenage women. It is likely that these symptoms are caused by female genital schistosomiasis and we suggest that simple patient history taking on risk water contact behaviour may serve as a diagnostic indicator when sophisticated diagnostic tools are unavailable. Further studies are needed to explore whether this approach may also be applicable for older women. Moreover, studies should be done in endemic settings to determine how to differentiate between STIs, cancer, and female genital schistosomiasis. Regular mass drug administration of Praziquantel should be offered to those at risk, particularly school-aged children and women.

## Figures and Tables

**Table 1:** Activity risk coefficient (ARC) for each water contact activity.

Activity	Time Range (h)	Median Body Surface (%)	Activity Risk Coefficient
Playing	1–3	100	5
Bathing	1–3	100	5
Washing blankets	3–5	35	4
Laundry	1–3	35	3
Fishing	1–3	35	3
Collecting water	≤1	20	1
Crossing	≤1	25	1

**Table 2:** Age distribution of the study participants.

Age	Number of Pupils (%)
16–17	214 (24.2)
18–19	397 (44.9)
20–22	272 (30.8)
Total	883

**Table 3:** Multivariable logistic regression analyses (MVA) of eight urogenital symptoms, total lifetime water contact score, and different causal agents in an *S. haematobium* endemic environment. Only genital infections exceeding a significance level of 15% were included in the MVA.

Symptom	Adjusted OR	95% CI	*p* Value ^a^
**Red urine**			
Water contact score ^b^	1.16	1.09–1.24	<0.001
*Neisseria gonorrhea* ^c^	1.65	1.07–2.55	0.023
**Stress incontinence**			
Water contact score ^b^	1.11	1.04–1.18	0.001
*Neisseria gonorrhea* ^c^	1.70	1.10–2.63	0.016
**Burning sensation in the genitals**			
Water contact score ^b^	1.09	1.03–1.15	0.005
*Neisseria gonorrhea* ^c^	1.83	1.19–2.81	0.006
**Spot bleeding** ^d^			
Water contact score ^b^	1.10	1.02–1.18	0.012
**Abnormal discharge smell**			
Water contact score ^b^	1.07	1.01–1.14	0.018
*Neisseria gonorrhea* ^c^	1.49	0.97–2.29	0.070
**Bloody discharge** ^d^			
Water contact score ^b^	1.11	1.02–1.21	0.020
**Lower abdominal pain** ^e^			
Water contact score ^b^	1.07	1.00–1.14	0.028
*Neisseria gonorrhea* ^c^	1.46	0.91–2.35	0.119
**Genital ulcer**			
Water contact score ^b^	1.08	1.00–1.16	0.038
*Neisseria gonorrhea* ^c^	1.63	1.01–2.64	0.045

^a^ Multivariable logistic regression; ^b^ Calculated by multiplying water contact frequency with an estimated activity risk coefficient and a lifetime exposure coefficient; ^c^ Strand displacement assay (ProbeTec CT/GC); ^d^ Excluded those using hormonal contraceptives; ^e^ Excluded those currently menstruating. OR: odds ratio.

**Table 4:** Multivariable logistic regression analyses of seven urogenital symptoms in those reporting current water contact and different causal agents in an *S. haematobium* endemic environment. Only genital infections exceeding a significance level of 15% were included in the MVA.

Symptom	Adjusted OR	95% CI	*p* Value ^a^
**Red urine**			
Water contact score ^b^	1.19	1.07–1.32	0.001
*Neisseria gonorrhoea* ^c^	1.46	0.75–2.83	0.266
**Pain on urination**			
Water contact score ^b^	1.15	1.04–1.27	0.006
*Neisseria gonorrhoea* ^c^	1.93	0.93–4.01	0.078
**Burning sensation in the genitals**			
Water contact score ^b^	1.17	1.06–1.30	0.002
*Neisseria gonorrhoea* ^c^	1.99	1.02–3.88	0.043
**Stress incontinence**			
Water contact score ^b^	1.17	1.05–1.30	0.004
*Neisseria gonorrhoea* ^c^	1.58	0.82–3.07	0.175
**Spot bleeding** ^d^			
Water contact score ^b^	1.18	1.05–1.33	0.005
Post coital bleeding ^d^			
**Water contact score** ^b^	1.18	1.04–1.34	0.012
Abnormal discharge smell			
Water contact score ^b^	1.11	1.01–1.22	0.027

^a^ Multivariable logistic regression; ^b^ Calculated by multiplying water contact frequency with an estimated activity risk coefficient and a lifetime exposure coefficient; ^c^ Strand displacement assay (ProbeTec CT/GC); ^d^Excluded those using hormonal contraceptives.
